# Chemiluminescent Deoxyribozyme Sensors for DNA-Editing Enzymes

**DOI:** 10.1021/acschembio.5c00927

**Published:** 2026-04-24

**Authors:** Martin Jakubec, Michal Svoboda, Jaroslav Kurfürst, Katerina Svehlova, Václav Veverka, Edward A. Curtis

**Affiliations:** † 89220 Institute of Organic Chemistry and Biochemistry of the Czech Academy of Sciences, Prague 166 10, Czech Republic; ‡ Department of Genetics and Microbiology, Faculty of Science, Charles University in Prague, Prague 128 44, Czech Republic; § Department of Informatics and Chemistry, University of Chemistry and Technology, Prague 166 28, Czech Republic; ∥ Department of Cell Biology, Faculty of Science, Charles University in Prague, Prague 128 44, Czech Republic

## Abstract

DNA-editing enzymes such as those in the APOBEC family of cytidine deaminases play important roles in both normal and pathogenic function, while engineered enzymes offer exciting new possibilities for genome editing. Despite their importance, widely used assays for DNA-editing enzymes are time-consuming and expensive. Here, we describe a new assay for DNA-editing enzymes in which the substrate in the reaction is a chemiluminescent deoxyribozyme called Supernova. Editing alters the sequence of Supernova, which results in a change in catalytic activity and light production. By analyzing a data set of Supernova variants previously identified by selection and high-throughput sequencing, it was possible to generate sensors with a wide range of specificities. Sensors were also developed for APOBEC3A, a cytidine deaminase which converts C to U in single-stranded DNA and RNA. These include a turn-off sensor that produces light 14-fold slower after incubation with recombinant APOBEC3A than in its absence, and a turn-on sensor that generates light 10-fold faster after incubation with APOBEC3A than in its absence. Assays that use these sensors are faster and less expensive than existing ones, and should be particularly useful for applications such as high-throughput screening.

## Graphical abstract

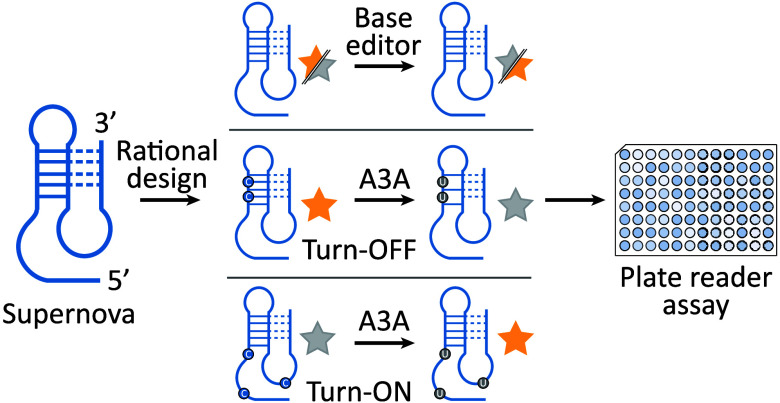

## Introduction

Enzymes that modify the sequences of nucleic acids play important roles in both normal and pathogenic biological function. One important class of such enzymes is the APOBEC family of cytidine deaminases, which change C to U in single-stranded DNA and RNA substrates.^1–5^ APOBEC enzymes mediate cellular immunity by editing viral DNA, but mutational patterns consistent with APOBEC have also been observed in many human cancers.^2–5^ Another important class of editing enzymes are adenine deaminases such as ADAR.^6^ These normally convert A to I in double-stranded RNA substrates, but can also modify DNA in the context of DNA/RNA hybrids. Programmed adenosine to inosine editing is thought to be a mechanism to increase protein diversity, but has also been linked to diseases such as the neurological Aicardi-Goutières syndrome^7^ and certain cancers.^8^ Although editing reactions in nature appear to be mostly limited to C to U and A to I conversions, artificial base editors with new specificities have also been developed using a variety of strategies.^9–11^ These enzymes make it possible to perform genome editing without DNA cleavage and therefore reduce the likelihood of generating pathogenic single and double-stranded DNA breaks relative to methods which use nucleases such as CRISPR-Cas9 or TALENs.

The importance of enzymes such as APOBEC3A and ADAR highlights the need for quick, easy, and inexpensive assays for DNA-editing reactions. Such assays can be used to optimize reaction conditions and characterize substrate specificities of DNA-editing enzymes, to search for new DNA-editing enzymes in biological materials such as cell extracts, and to detect the presence of existing enzymes in diagnostic samples. Moreover, if such assays can be scaled-up, they can also be used in high-throughput screens to identify small molecules that modulate the activities of DNA-editing enzymes. The development of small molecule inhibitors is particularly important in the case of APOBEC enzymes, which have been linked to many types of human cancers.^2–5^ Despite their importance, widely used assays for DNA-editing enzymes are generally expensive and require multiple steps. In the standard assay for APOBEC3A,^12^ for example, the enzyme is first incubated with a DNA substrate that contains a covalently linked fluorophore (or FRET donor) at one end and a covalently linked quencher (or FRET acceptor) at the other. After conversion from C to U, the substrate is incubated with the enzyme uracil-DNA glycosylase (UDG) to remove the newly created U at the reaction site. The resulting abasic site is then cleaved with base, which separates the fluorophore from the quencher (or the FRET donor from the acceptor) and leads to an increase (or decrease) in fluorescence.

To address the need for improved assays, in this study, we set out to develop chemiluminescent sensors for DNA-editing enzymes based on Supernova, a light-producing deoxyribozyme recently developed in our group.^13^ One general advantage of Supernova-based assays is that they are quick and easy: a detectable signal can be generated in minutes by simply mixing Supernova with buffer and substrate and putting the reaction into a plate reader. Another is that high signal-to-noise ratios can be achieved at low costs. For example, signal-to-noise ratios greater than 100-fold can be achieved at a cost of less than €0.1 per reaction, and signal-to-noise ratios approaching 10,000-fold can be attained at a cost of less than €1 per reaction.^14^ The idea of using Supernova as a sensor for DNA-editing enzymes is also appealing because Supernova itself can act as a substrate for such enzymes without additional modification. Here, we show that conservation patterns in a data set of Supernova variants previously identified by selection and high-throughput sequencing^13^ can be used to develop chemiluminescent sensors with a wide range of specificities. Sensors were also developed for APOBEC3A, a cytidine deaminase which converts C to U in single-stranded DNA and RNA substrates. Examples include a turn-off sensor inhibited 14-fold and a turn-on sensor activated 10-fold by recombinant APOBEC3A. These sensors use a workflow that is rapid, simple, and inexpensive, and we anticipate that they will facilitate study of DNA-editing proteins on many different levels.

## Materials and Methods

### Oligonucleotides

All oligonucleotides used in this study were ordered from Eurofins Genomics and purified by HPLC or PAGE before use. For the sequences of oligonucleotides, see Table S1.

### Sensor Design

Sensors were designed using a previously described data set of Supernova variants.^13^ This was generated by randomly mutagenizing our initial isolate of Supernova (called H1) at a rate of 21% per position and identifying the catalytically active variants in this library using *in vitro* selection and high-throughput sequencing. By analyzing conservation patterns in this data set, it was possible to design sensors that are turned off or on by specific mutational changes (such as those induced by DNA-editing enzymes). For example, because Supernova contains several conserved GG motifs (Figure S1), it can in principle be used as a turn-off sensor to detect a hypothetical DNA-editing enzyme that converts GG to another sequence (such as CG). Similarly, a Supernova variant in which all conserved GG sites are replaced with (for example) CG can in principle be used as a turn-on sensor to detect a hypothetical DNA-editing enzyme that converts CG to GG. To design Supernova-based APOBEC3A sensors, sequences in this data set were scanned for dinucleotides at which the frequency of TC (the preferred substrate of APOBEC3A) was significantly different than the frequency of TT (which we hypothesized would in some cases behave similarly to TU, which is the product of the APOBEC3A reaction but was not encoded by our library). Candidate sensors identified in this way were synthesized as pairs in which one sequence contained TC and the other contained TU at the expected APOBEC3A reaction site. Both variants in each pair were then tested for their ability to catalyze a chemiluminescent reaction under optimized reaction conditions. Sequences in which the fold difference between the initial rate of light production of the TC variant and the TU variant was largest were then tested for responsiveness to recombinant APOBEC3A. In each case, the initial rate of light production by the TC variant after incubation with APOBEC3A was compared to the initial rate of light production in the absence of APOBEC3A. The best sensors were further optimized by rational design. In the case of turn-off sensors, this typically involved adding additional TC sites to the sequence to further reduce activity of the edited version. In the more complicated case of turn-on sensors, this involved both the introduction of new TC sites (to further reduce the signal produced by unedited variants) and the removal of existing TC sites (to minimize the likelihood of off-target APOBEC3A reactions that could reduce the activity of edited sequences).

### APOBEC3A Detection

In a typical reaction, sensor was diluted into Milli-Q water, heated at 65 °C for 2 min, and cooled at RT for 5 min. Recombinant APOBEC3A in 2× buffer was then added to initiate the DNA-editing reaction. Final concentrations were 50 nM APOBEC3A, 20 mM NaCl, 0.5% Triton, 7.5 mM Tris–HCl pH 7.4, and 3.3 μM DNA in a total volume of 30 μL. Reactions were incubated at RT in the dark with moderate shaking for 20 min. APOBEC3A was inactivated by light vortexing. Following the addition of Supernova buffer (final concentrations were typically 650 μM ZnCl_2_, 20 mM KCl, 20 mM Tris pH 7.4, and 1 μM DNA, as well as diluted components from the previous buffer), the light-producing reaction was initiated by adding CDP-Star to a final concentration of 62.5 μM in a final volume of 100 μL.

### Analysis of Light Production

Reactions were analyzed by pipetting 100 μL of the reaction sample into the wells of half-area flat-white 96-well microplates (Corning). Light production was measured for 1 h using a Spark Microplate Reader from Tecan. Samples containing all components except Supernova and CDP-Star (buffer controls) were used to measure background. To prevent extreme numerical values obtained by subtracting two numbers of very similar values at early time points, the baseline light production was linearized by dividing the cumulative light production after 1 h by the number of time points measured by the plate reader. Light production from spontaneous CDP-Star breakdown was measured by subtracting the linearized amount of light produced in reactions containing buffer alone from the amount produced in reactions containing CDP-Star and buffer but no Supernova (Figure S2). Similarly, light production from deoxyribozyme catalyzed reactions was measured by subtracting the linearized amount of light produced in reactions containing buffer alone from the amount produced in reactions containing CDP-Star, buffer, and Supernova (Figure S2). The RLU value at the zero point of each reaction was subtracted from all time points prior to curve fitting. Light production initially increased linearly with time in both nonenzymatic and Supernova-catalyzed reactions, and initial rates of light production reported in this manuscript (RLUs per minute) correspond to the slope of this line in the first 5 min of the reaction. In some cases, signal-to-noise rations were also determined. These were calculated by dividing the cumulative light production of Supernova-catalyzed reactions at every time point by the cumulative light production of reactions containing only CDP-Star and buffer at the same time point. Maximum signal-to-noise ratios reported in this study correspond to the maximum values observed in 1 h time courses. Supernova was used as a positive control in all experiments. See ref 14 for additional details.

**Figure 1:**
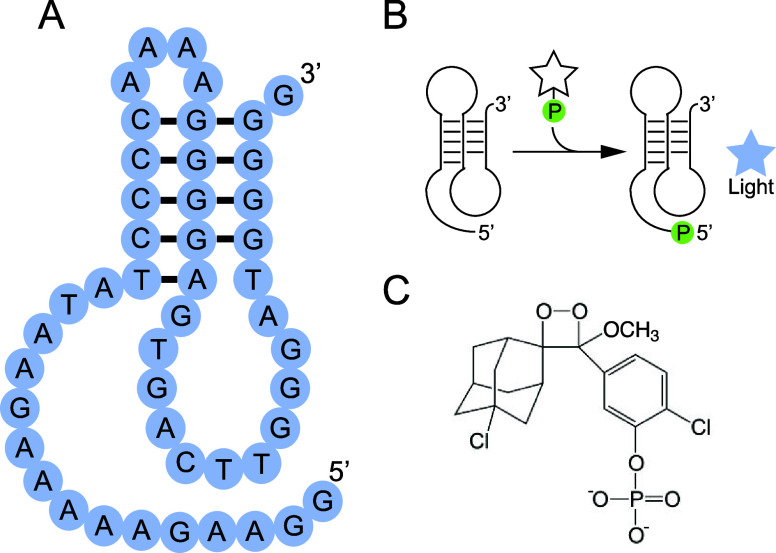
Light production using Supernova. (A) Secondary structure model of Supernova. (B) Simplified mechanism of light production by Supernova. (C) Chemical structure of CDP-Star, the substrate used by Supernova.

**Figure 2:**
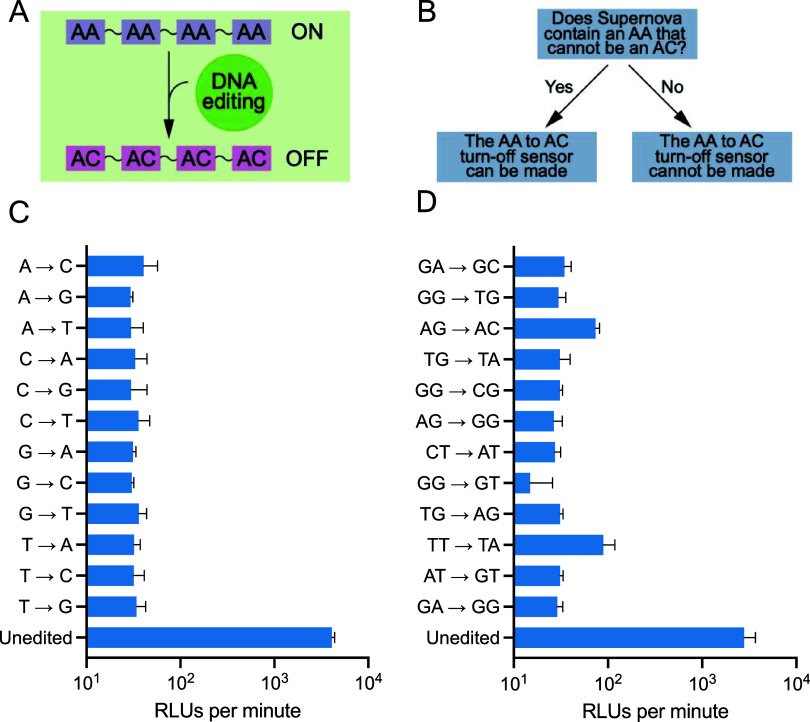
Turn-off sensors for hypothetical DNA-editing enzymes. (A) Simplified mechanism of a turn-off sensor based on Supernova. (B) Design of turn-off sensors using knowledge of the sequence requirements of Supernova.^13^ (C) Biochemical characterization of turn-off sensors for hypothetical DNA-editing enzymes that catalyze each of the 12 possible reactions in which all examples of a specific nucleotide in a sequence (such as A) are changed to another nucleotide (such as C). (D) Biochemical characterization of turn-off sensors for hypothetical DNA-editing enzymes that catalyze reactions in which all examples of a specific dinucleotide in a sequence (such as AG) are changed to a single-mutation variant (such as GG). Each column shows the average value of three independent experiments, and error bars represent 1 standard deviation. Reactions contained 1 μM Supernova and 62.5 μM CDP-Star in a buffer containing 650 μM ZnCl_2_, 20 mM KCl, and 20 mM HEPES pH 7.4. Cumulative light production was measured for 1 h, and the initial rate of light production was determined from time points in the first 5 min of each reaction.

**Figure 3:**
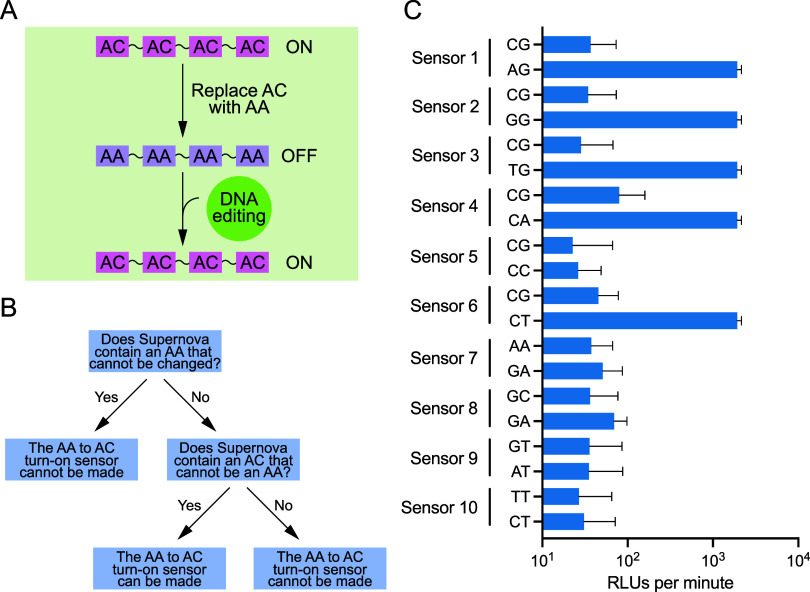
Turn-on sensors for hypothetical DNA-editing enzymes. (A) Simplified mechanism of a turn-on sensor based on Supernova. (B) Design of turn-on sensors using knowledge of the sequence requirements of Supernova.^13^ (C) Biochemical characterization of turn-on sensors for hypothetical DNA-editing enzymes that catalyze reactions in which all examples of a specific dinucleotide in a sequence (such as CG) are changed to a single-mutation variant (such as AG). Each column shows the average value of three independent experiments, and error bars represent 1 standard deviation. Reactions contained 1 μM Supernova and 62.5 μM CDP-Star in a buffer containing 650 μM ZnCl_2_, 20 mM KCl, and 20 mM HEPES pH 7.4. Cumulative light production was measured for 1 h, and the initial rate of light production was determined from time points in the first 5 min of each reaction.

**Figure 4:**
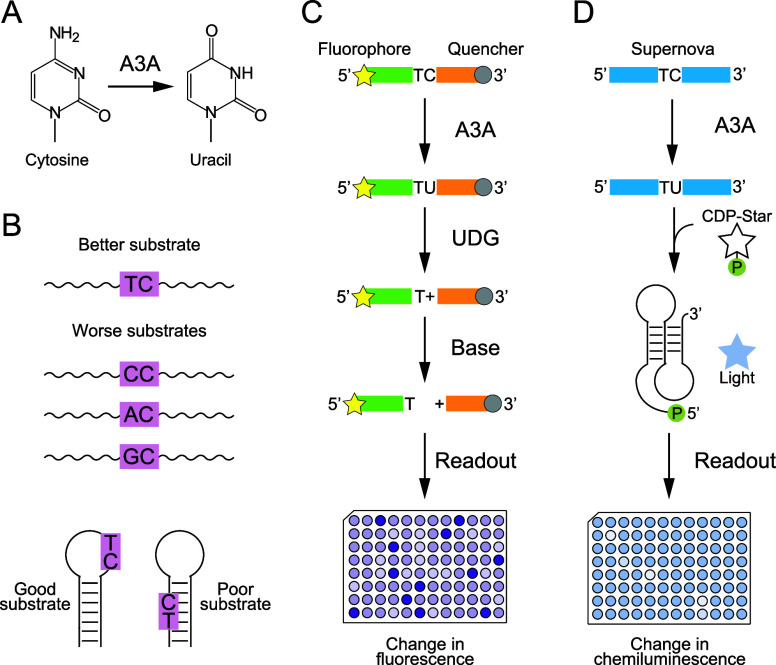
DNA editing by APOBEC3A. (A) APOBEC3A converts C to U in DNA and RNA (B) APOBEC3A edits TC sites in single-stranded DNA more efficiently than CC, AC, or GC sites, preferentially modifies cytosines in short loops of stable hairpins, and does not efficiently edit double-stranded DNA. (C) Standard assay for APOBEC3A activity. This assay requires the protein enzyme UDG as well as an oligonucleotide substrate containing a fluorophore (or FRET donor) at one end and a quencher (or FRET acceptor) at the other. (D) Faster and less-expensive Supernova-based assay for APOBEC3A activity described in this study.

**Figure 5:**
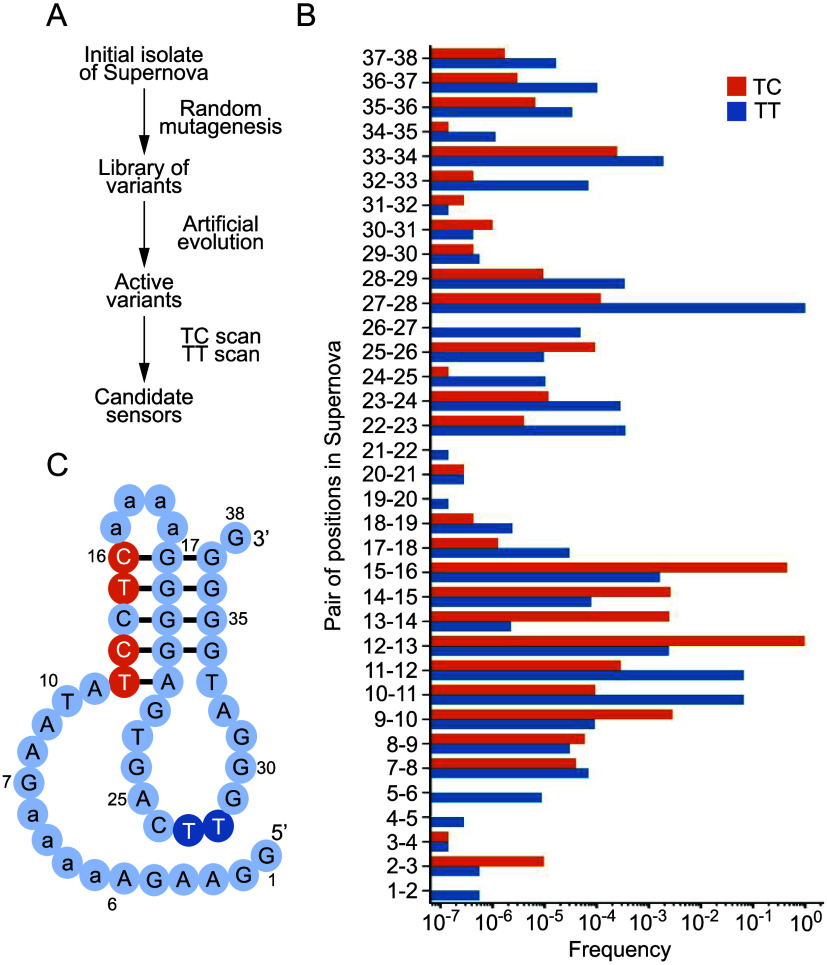
Design of turn-off and turn-on sensors for APOBEC3A. (A) Workflow to identify APOBEC3A sensors. (B) TC and TT frequencies at each dinucleotide in a previously described data set of Supernova variants.^13^ (C) Secondary structure model of the H1 core variant of Supernova.^13^ Positions at which the frequency of TC is significantly higher than that of TT are shown in orange and can be used to design turn-off sensors. Positions at which the frequency of TT is significantly higher than that of TC are shown in blue, and can be used to design turn-on sensors. Positions with bases shown in lowercase font correspond to those in AAAA spacers that were introduced in minimization experiments^13^ and are not numbered.

**Figure 6:**
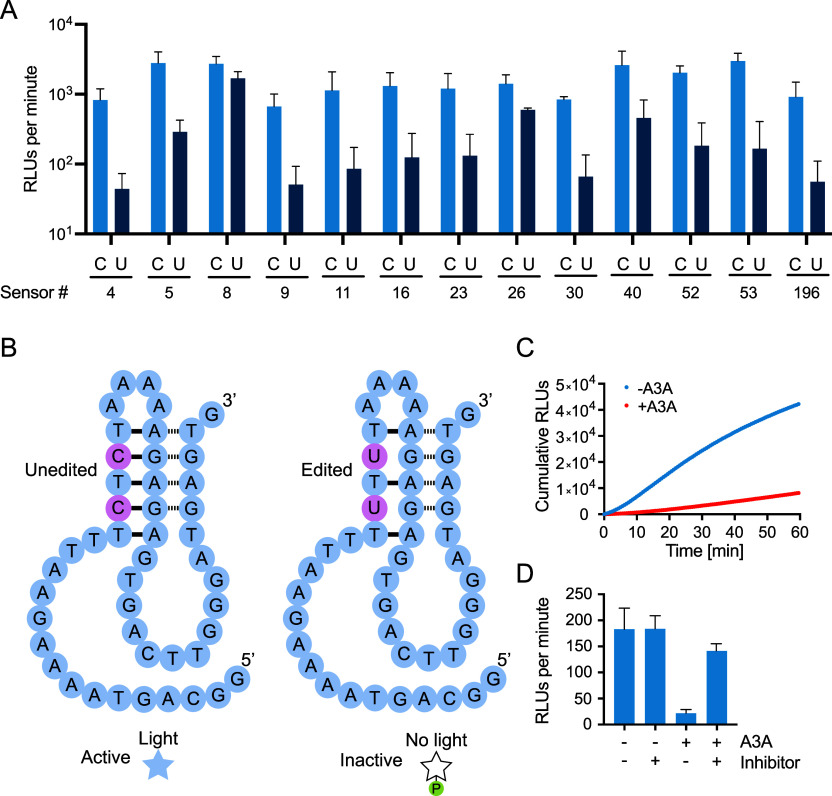
APOBEC3A turn-off sensors. (A) Light production of 13 candidate APOBEC3A turn-off sensors that contain either TC (light bars) or TU (dark bars) at different target sites. Reactions contained 1 μM Supernova and 62.5 μM CDP-Star in a buffer containing 650 μM ZnCl_2_, 20 mM KCl, and 20 mM HEPES pH 7.4. Cumulative light production was measured for 1 h, and the initial rate of light production was determined from time points in the first 5 min of each reaction. (B) Secondary structure model of an APOBEC3A turn-off sensor called Sensor 4. (C) Total light production of Sensor 4 as a function of time following incubation in the absence or presence of APOBEC3A. (D) Inhibition of this sensor by recombinant APOBEC3A. Sensor inhibition does not occur when the sensor is preincubated with both APOBEC3A and aurintricarboxylic acid (a nonspecific enzyme inhibitor). Reactions containing 3.3 μM Supernova and 50 nM APOBEC3A were incubated in a buffer containing 20 mM NaCl, 0.5% Triton, 7.5 mM Tris-HCl pH 7.4, and 0 or 30 μM inhibitor for 20 min. After adding CDP-Star and Supernova buffer (final concentrations were 1 μM Supernova, 15 nM APOBEC3A, 62.5 μM CDP-Star, 650 μM ZnCl_2_, 20 mM KCl, and 20 mM Tris-HCl pH 7.4, as well as diluted components from the previous buffer), reactions were put into a plate reader. Cumulative light production was measured for 1 h, and the initial rate of light production was determined from time points in the first 5 min of the reaction. Each column shows the average value of three independent experiments, and error bars represent 1 standard deviation.

### UDG Cleavage

Sensors were diluted into Milli-Q water, heated at 65 °C for 2 min and cooled at RT for 5 min. Recombinant APOBEC3A in 2× buffer was then added to initiate the DNA-editing reaction. Final concentrations were 50 nM APOBEC3A, 20 mM NaCl, 0.5% Triton, 7.5 mM Tris–HCl pH 7.4, and 3.3 μM DNA in a total volume of 30 μL. Reactions were incubated at RT in the dark with moderate shaking for 20 min. The reaction was then stopped by adding 1 mM EDTA Tris pH 8.5 until the reaction pH reached 8.1. UDG was added at a concentration of 1 U/sample, followed by incubation at 37 °C for 30 min. Abasic sites were hydrolyzed by adding 100 mM NaOH and incubating at 80 °C for 20 min. Samples were then analyzed by PAGE on a 15% denaturing gel.

### APOBEC3A Expression

Expression and purification of APOBEC3A was based on a previously described protocol.^15^ An expression plasmid containing *Escherichia coli* optimized MBP_APOBEC3A_6×His was transformed into C43­(DE3) pLysS *E. coli* cells. Colonies were inoculated into 50 mL of LB including Kanamycin/Chloramphenicol/1% Glucose and grown overnight at 37 °C. Larger cultures of 4 L were then inoculated with a 1:100 dilution of an overnight culture and grown at 37 °C until ODs reached ∼0.6, at which point MgSO_4_ and ZnSO_4_ were added to the final concentrations of 20 μM and 10 μM, respectively. Expression of MBP_APOBEC3A_6×His was induced with 0.25 mM IPTG and cultures were grown for 3 h at 37 °C with shaking. An inactive mutant of APOBEC3A used in control experiments contained the E72A mutation. Cells were pelleted at 6000*g* for 15 min, resuspended in 25 mL of wash buffer (50 mM Tris pH 7.5, 150 mM NaCl, 10% glycerol, 25 mM imidazole) per liter of culture, and EDTA-free protease inhibitor tablets, Benzonase, and an antifoam agent were added. Cells were lysed by Dounce homogenizer disruption followed by three passages through a CF1 microfluidizer. Cellular debris was removed by centrifugation at 20,000*g* for 30 min. Lysate was filtered through a 0.6 μm Millipore syringe filter. A NiNTA purification at 4 °C was then performed. Ten column volumes of wash buffer (50 mM Tris pH 7.5, 150 mM NaCl, 10% glycerol, 25 mM imidazole) were then run through the column. Four bound fractions were eluted using elution buffers (50 mM Tris pH 7.5, 150 mM NaCl, 10% glycerol, and either 150 mM imidazole for the first fraction or 400 mM imidazole for the remaining fractions). Elutes were analyzed on 10% SDS-PAGE gels stained with Coomassie Blue with the fusion products corresponding to ∼70 kDa. Eluted fractions were dialyzed into TEV reaction buffer (50 mM Tris pH 8.0, 0.5 mM EDTA, 1 mM DTT) at 4 °C for two 3-h-long rounds of dialysis. TEV was added to a final concentration of 40 μg/mL and dialyzed overnight with no mechanical movement. The effectivity of TEV cleavage was assessed on a 15% SDS-PAGE gel. Precipitate was removed by 10 min of centrifugation at 20,000*g* at 4 °C, and the product was purified via AKTA FPLC using a 5 mL heparin column (GE HiTrap) and flow rates of 0.5–1 mL/min. The heparin column was equilibrated with 10 column volumes of wash buffer (50 mM Tris pH 8.0, 0.5 mM EDTA, 1 mM DTT), and samples were loaded in 10 column volumes. Fractions were collected using a gradient of elution buffer (50 mM Tris pH 8.0, 0.5 mM EDTA, 1 mM DTT, 1 M NaCl) over 15 column volumes. Eluted fractions containing the ∼23 kDa APOBEC3A were dialyzed overnight into dialysis buffer (50 mM Tris pH 7.5, 50 mM NaCl, 10% glycerol, 0.5 mM DTT) and filtered using an equilibrated Superdex 75 10/300 Gel filtration column on an AKTA FPLC system. Protein concentrations were adjusted to 1 mg mL^–1^, and Tween-20 was added to the final concentration of 0.01%. Samples were aliquoted and stored at −80 °C.

## Results

### Chemiluminescent Turn-Off Sensors for Hypothetical DNA-Editing Enzymes

Supernova is a light-producing deoxyribozyme recently discovered in our group.^13^ It generates a chemiluminescent signal by transferring the phosphate group from the 1,2-dioxetane substrate CDP-Star to its own 5′-hydroxyl group, which triggers a decomposition reaction and a flash of blue light (Figure 1). Other deoxyribozymes generate fluorescent^16,17^ or colorimetric signals using a similar mechanism.^18^ Supernova can be modified so that it only generates light in the presence of a target molecule,^13^ and identification of additional architectures for chemiluminescent deoxyribozyme sensors is an active area of research in our group. The initial goal of this study was to develop variants of Supernova that are inactivated by specific mutational changes (such as those catalyzed by DNA-editing enzymes). In principle, it should be possible to develop a turn-off sensor for any DNA-editing enzyme that modifies a conserved sequence element in Supernova. Such a sensor would generate light in the absence of the DNA-editing enzyme but be inactivated by the DNA-editing reaction (Figure 2a). To identify conserved sequence motifs that could potentially be used as substrates by such enzymes, we analyzed a Supernova library that was generated by randomly mutagenizing the sequence of a single variant of Supernova called H1 at a rate of 21% per position. Active variants were previously isolated from this library using *in vitro* selection and characterized using high-throughput sequencing.^13^ Conservation patterns in this data set were used to identify potential recognition sites for different types of DNA-editing enzymes (Figures 2b and S1).

We initially focused on sensors for hypothetical editing enzymes with single nucleotide recognition sites. Twelve types of editing reactions are possible (A to C, A to G, A to T, C to A, C to G, C to T, G to A, G to C, G to T, T to A, T to C, and T to G), and analysis of conserved positions in Supernova suggested that it should be possible to construct turn-off sensors for hypothetical DNA-editing enzymes with each of these specificities (Figure S1). To confirm this, the effects of each of these 12 types of mutations were tested in the context of an optimized variant of Supernova. For each type of sensor, all positions at which the substrate nucleotide occurred (for example, A) were changed to its product (for example, C) to simulate a complete editing reaction. Each sensor functioned as expected, and fold inhibition values (defined as the initial rate of light production of the unmodified sensor divided by the initial rate of a completely edited version of the sensor) ranged from 97-fold to 139-fold under these conditions (Figure 2c). Because Supernova is inhibited by each of these 12 types of changes, such sensors would not be specific for a particular editing reaction. However, they could facilitate rapid characterization of purified DNA-editing enzymes with known specificities as well as identification of inhibitors in high-throughput screens.

We next investigated the possibility of developing sensors for hypothetical DNA-editing enzymes that recognize dinucleotides. Sixteen different dinucleotide recognition sites (such as GA) are possible, and each can be mutated to one of six single-mutation variants (such as GA to AA, CA, TA, GC, GG, or GT). This means that 96 different reactions can occur. Analysis of conservation patterns in Supernova showed that it contains five almost invariant dinucleotides: AC, AG, GA (two times), GG (three times), and TG (two times) (Figure S1). These can be used to generate turn-off sensors for 30 of the 96 possible reactions. When constrained positions are also considered, plausible turn-off sensors can be constructed for many of the 66 remaining reactions. For example, because positions two and three in Supernova must be either GA or GC, they can be used to make turn-off sensors for hypothetical editing enzymes that convert GA to AA, CA, TA, GG, or GT as well as those that convert GC to AC, CC, TC, GG, or GT. Twelve randomly chosen examples of such sensors were tested. Each functioned as expected, and fold inhibition values (defined as before as the initial rate of light production of the unmodified sensor divided by the initial rate of a completely modified version of the sensor) ranged from 31-fold to 175-fold (Figure 2d). These results show that it is possible to construct Supernova-based turn-off sensors for a wide range of hypothetical DNA-editing enzymes.

### Chemiluminescent Turn-On Sensors for Hypothetical DNA-Editing Enzymes

We next considered the more complicated case of turn-on sensors. Such a sensor could in principle be constructed by generating an inactive version of Supernova that contains a mutation in a conserved sequence element. This could then be activated by a DNA-editing enzyme that corrects the mutation. Development of such turn-on sensors is more difficult than that of turn-off sensors because the reaction catalyzed by the DNA-editing enzyme must satisfy two conditions: it must correct the mutated site, and at the same time must not mutate a conserved sequence element in another part of Supernova. However, an advantage of such sensors is that they are generally more specific than turn-off sensors. Analysis of conservation patterns in Supernova revealed that this type of approach could not be used to develop turn-on sensors for hypothetical DNA-editing enzymes that convert all examples of a specific nucleotide (such as A) into another nucleotide (such as C). The reason is that Supernova contains at least one position that must be A, at least one position that must be C, at least one position that must be G, and at least one position that must be T (Figure S1). However, because not every possible dinucleotide motif occurs in Supernova, it was possible in some cases to design turn-on sensors for hypothetical DNA-editing enzymes that recognize dinucleotides (Figure 3a,b). For example, by mutating the conserved AG motif at positions 4 and 5 in Supernova to CG (which does not occur as a conserved sequence element in Supernova; Figure S1), it was possible to generate a turn-on sensor for a hypothetical DNA-editing enzyme that converts CG to AG. Turn-on sensors for at least 10 of the 96 possible reactions in which a dinucleotide recognition site is converted to a single-mutation variant appeared to be possible based on the conservation patterns in Supernova. Five of these turn-on sensors functioned as expected, and fold activation values (defined here as the initial rate of light production of the completely edited version of the sensor divided by the initial rate of the unmodified version of the sensor) ranged from 21-fold to 53-fold (Figure 3c). Such sensors are more specific than our turn-off sensors because they are only activated by a single DNA-editing reaction, whereas the turn-off sensors can be inhibited by many different editing reactions. For this reason, these turn-on sensors could be useful for discovery and characterization of DNA-editing enzymes in complex biological samples as well as detection of known DNA-editing enzymes in diagnostic tests. Taken together, these experiments demonstrate that Supernova can be used to develop turn-on sensors for DNA-editing enzymes.

### Turn-Off Sensors for the Detection of APOBEC3A

We next investigated whether this approach can be extended to existing DNA-editing enzymes. We focused our attention on APOBEC3A,^1–5^ a DNA-editing enzyme that converts C to U in DNA and RNA (Figure 4). As previously noted, the standard assay for APOBEC3A is expensive and time-consuming, and we hypothesized that a Supernova-based assay would be cheaper and faster (Figure 4c,d). Previous studies using single-stranded DNA suggest that TC is the preferred editing site of APOBEC3A,^19,20^ although CC,^21,22^ and even AC^22^ can be efficiently modified in certain sequence contexts (Figure 4b). Flanking nucleotides can affect reactivity,^20,21^ and structural context is also important: C residues in DNA duplexes are not efficiently edited,^21,22^ while those in short loops of stable hairpins are highly reactive (Figure 4b).^2,5^ Based on these studies, we hypothesized that TC motifs in Supernova would be good substrates for APOBEC3A, especially if Supernova was not folded, and that TC to TU changes would in some cases modulate Supernova activity. Because U was not encoded in our previously characterized Supernova library, we instead searched our data set of variants for positions at which TC can occur but TT (which we thought in some cases would behave similarly to TU) cannot. This revealed several positions at which TC to TU modification was expected to result in inactivation of Supernova (Figure 5). To confirm the effects of these mutations, 13 candidate sensors were synthesized. In each case, one variant of the sensor was synthesized that contained TC at target sites (corresponding to the substrate) and another was synthesized that contained TU at these sites (corresponding to the expected product). Each of these sensors worked to some extent, including 11 examples for which the initial rate of light production was at least 5-fold faster for the TC variant than for the TU variant (Figure 6a). We next investigated whether these sensors could be inactivated by recombinant APOBEC3A. To increase the likelihood that target sites would be available for modification, APOBEC3A was initially incubated with Supernova in a buffer that did not contain zinc. Under these conditions, Supernova cannot fold into its catalytically active conformation,^13,14^ and we hypothesized that this would make TC sites more accessible to APOBEC3A. After a short incubation to allow TC to TU editing to occur (note that APOBEC3A is active in this buffer because the zinc ions needed for its function bind to the enzyme during expression and remain bound during purification), zinc and CDP-Star were added to the reaction, and light production was measured in a plate reader. The initial rate of light production of the best of these turn-off sensors, called Sensor 4, was 14-fold slower after incubation with recombinant APOBEC3A than in its absence (Figures 6b,c and S3). The rate of light production of the sensor did not change when it was incubated with a catalytically inactive mutant of APOBEC3A (Figure S4a), suggesting that inhibition is due to editing rather than a nonspecific effect of the protein. Analysis of the sensor using the standard UDG assay (which detects C to U changes) provided additional evidence that the sequence of this sensor is changed in the reaction (Figure S4b). The sensor could also distinguish reactions that contained the nonspecific APOBEC3A inhibitor aurintricarboxylic acid from those that did not (Figure 6d), raising the possibility that it can be used to identify new inhibitors in high-throughput screens. These results show that our approach can be used to construct turn-off sensors for APOBEC3A.

### Turn-On Sensors for the Detection of APOBEC3A

Analysis of conservation patterns in Supernova also revealed several possible ways to construct turn-on sensors that are activated rather than inhibited by APOBEC3A (Figure 5). This was facilitated by the purine-rich nature of Supernova, a fortuitous consequence of which is that Supernova does not contain a single conserved TC motif. However, several positions in the sequence were identified at which TT (and possibly TU) can occur but TC cannot. To determine the extent to which these sensors functioned as expected, five examples were synthesized in pairs in which one variant contained TC and the other contained TU at the target site or sites. While the TC and TU variants of four of these candidate sensors had similar activities, the initial rate of light production of a fifth one, called Sensor 11/28, was 20.1-fold higher as the TU variant than as the TC variant (Figure 7a). This sensor was also activated by recombinant APOBEC3A: its initial rate of light production was 10-fold faster after preincubating with APOBEC3A than with buffer alone (Figures 7b,c and S5). As was the case for our turn-off sensor, this turn-on sensor was not activated by catalytically inactive APOBEC3A (Figure S6a), contained C to U mutations after incubation with APOBEC3A (Figure S6b), and could distinguish reactions that contained a nonspecific APOBEC3A inhibitor from those that did not (Figure 7d). Additional experiments revealed that, in some cases, turn-on sensors in which TC motifs were replaced by TU motifs (corresponding to the expected products of editing reactions) were inhibited upon further incubation with APOBEC3A (Figure S7a). This could indicate that initial editing of a sequence such as TCC generates a TUC site, which is then further edited to TUU in a chain reaction (Figure S7b). However, it could also reflect editing of noncanonical (non-TC) sites in Supernova such as CCCC.^1,22^ Taken together, these results show that our approach can be used to construct turn-on sensors for APOBEC3A.

**Figure 7:**
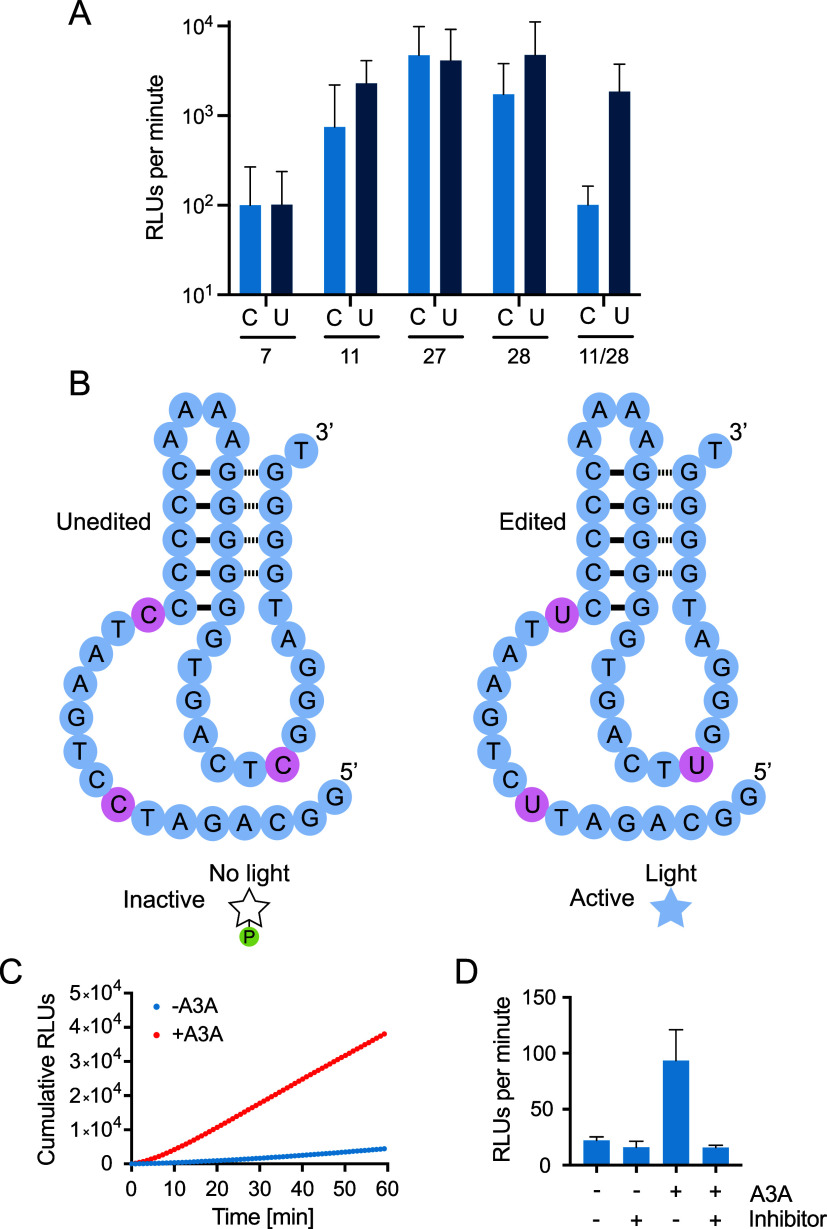
APOBEC3A turn-on sensors. (A) Light production of 5 candidate APOBEC3A turn-on sensors that contain either TC (light bars) or TU (dark bars) at different target sites. Reactions contained 1 μM Supernova and 62.5 μM CDP-Star in a buffer containing 650 μM ZnCl_2_, 20 mM KCl, and 20 mM HEPES pH 7.4, and the initial rate of light production was determined from time points in the first 5 min of the reaction. (B) Secondary structure model of an APOBEC3A turn-on sensor called 11/28. (C) Total light production of Sensor 11/28 as a function of time following incubation in the absence or presence of APOBEC3A. (D) Activation of Sensor 11/28 by recombinant APOBEC3A. Sensor activation does not occur when the sensor is incubated with both APOBEC3A and aurintricarboxylic acid (a nonspecific enzyme inhibitor). Reactions containing 3.3 μM Supernova and 50 nM APOBEC3A were incubated in a buffer containing 20 mM NaCl, 0.5% Triton, 7.5 mM Tris–HCl pH 7.4, and 0 or 30 μM inhibitor for 20 min. After adding CDP-Star and Supernova buffer (final concentrations were 1 μM Supernova, 15 nM APOBEC3A, 62.5 μM CDP-Star, 650 μM ZnCl_2_, 20 mM KCl, and 20 mM Tris-HCl pH 7.4, as well as diluted components from the previous buffer), reactions were put into a plate reader. Cumulative light production was measured for 1 h, and the initial rate of light production was determined from time points in the first 5 min of the reaction. Each column shows the average value of three independent experiments, and error bars represent 1 standard deviation.

### Optimization of Reaction Conditions and Sensor Performance

To further improve sensor performance, we explored the effects of different reaction conditions on light production. Consistent with previous studies,^13,14^ these sensors required zinc with an optimal concentration between 400 μM and 800 μM, and activity decreased significantly at higher or lower concentrations (Figure S8). Initial rates of activated sensors were highest when DNA-editing reactions were performed at pH 7.4, although comparable values were observed at lower pH values (Figure S9). Inactivation of turn-off sensors could be achieved using APOBEC3A concentrations as low as 30 nM for turn-on sensors and 20 nM for turn-off sensors (Figure S10), and under these conditions, virtually complete modification of Supernova could typically be achieved in a 10 min incubation (Figure S11). The presence of PEG in reactions also enhanced sensor performance (Figure S12). Control experiments confirmed that, as is the case for Supernova, a stable triple helix was required for light production (Figure S13). Taken together, these experiments indicate that high signal-to-noise ratios can be generated using low APOBEC3A concentrations, short incubation times, and a protocol without washes or purifications.

## Discussion

Deoxyribozymes are useful tools for a variety of applications.^23–33^ Although typically not as efficient as protein enzymes from the perspective of catalytic efficiency (but see also refs. 34,35 ), deoxyribozymes are easier and less expensive to synthesize, and can be more readily isolated and modified using the power of artificial evolution. We recently developed a chemiluminescent deoxyribozyme called Supernova,^13^ and, in recent work, have focused on the development of new assays that use this catalytic motif. In this study, we developed Supernova-based homogeneous assays that can be used to detect the activities of hypothetical DNA-editing enzymes with a wide range of specificities. These include assays for each of the 12 possible reactions in which all occurrences of a specific nucleotide in a sequence (such as A) are converted to another (such as C) and for some of the 96 possible reactions in which all instances of a specific dinucleotide (such as AG) are converted to a single-mutation variant (such as GG). We also demonstrate that such sensors can be used to detect the catalytic activity of APOBEC3A, a cytidine deaminase that converts C to U in single-stranded DNA and RNA. Furthermore, we show that our sensors can distinguish reactions that contain an APOBEC3A inhibitor from those that do not, raising the possibility that this approach can be used to identify new APOBEC3A inhibitors in high-throughput screens. Our assay offers a number of advantages relative to that typically used in the field.^12^ For one thing, it only requires CDP-Star and unmodified DNA, while the standard assay requires a doubly labeled DNA substrate as well as recombinant uracil-DNA glycosylase (UDG) protein. Both the substrate and UDG are expensive, and in our hands, the cost of this new assay is approximately 5.5-fold lower than that of the existing one. Our workflow is also approximately 3 times faster (Figure 4c,d). This is because in the standard assay, reaction products must first be incubated with UDG followed by base to cleave the edited site and separate the fluorophore (or FRET donor) from the quencher (or FRET acceptor). In our assay, however, reactions can be assayed directly after a short incubation with APOBEC3A. We also note that, after preparation of master mixes, our protocol requires only two pipetting steps per reaction (one in which APOBEC3A and buffer is added to Supernova to initiate DNA editing, and a second in which CDP-Star and Supernova buffer is added to edited Supernova to initiate light production). Such features are especially important for applications such as high-throughput screening in which many reactions need to be processed in parallel.^36^ However, our assay could also be useful for other applications. These include the identification of new DNA-editing enzymes in cell extracts and the discovery of DNA-editing proteins with new specificities in directed evolution experiments. From this perspective, we anticipate that the sensors described here will facilitate study of DNA-editing proteins on many different levels.

## Supplementary Material



## Notes

Sequences of all deoxyribozymes used in this study as well as additional experimental details (PDF)
