# Supplementary material for: Chemiluminescent Deoxyribozyme Sensors for DNA-Editing Enzymes
Source: ACS Chem Biol. 2026 Apr 24;21(5):941–50. doi: 10.1021/acschembio.5c00927 (PMC13184990; doi:10.1021/acschembio.5c00927)
Supplement: Supplementary file 1 [file cb5c00927_si_001.pdf]

Chemiluminescent deoxyribozyme sensors for DNA-editing enzymes

Martin Jakubec[1,2], Michal Svoboda[1], Jaroslav Kurfürst[1,3], Katerina Svehlova[1], Václav Veverka[1,4], and Edward A. Curtis[1*]

[1]Institute of Organic Chemistry and Biochemistry of the Czech Academy of Sciences, Prague 166 10, Czech Republic

[2]Department of Genetics and Microbiology, Faculty of Science, Charles University in Prague, Prague 128 44, Czech Republic

[3]Department of Informatics and Chemistry, University of Chemistry and Technology, Prague 166 28, Czech Republic

[4]Department of Cell Biology, Faculty of Science, Charles University in Prague, Prague 128 44, Czech Republic

Phone: +420 733 169 654
E-mail: curtis@uochb.cas.cz

*Corresponding author

Table S1. Sequences of all oligonucleotides used in this study.

	Sequence (5' → 3')
Supernova (H1)	GGA AGA AAA AGA ATA TCC CCA AAA GGG GAG TGA CTT GGG ATG GGG G
Pseudo-Hypernova	GGC AGA TCC TGA ATA TCT CCA AAA GGA GAG TGA CTT GGG ATG AGG T
A → C	GGC CGC TCC TGC CTC TCT CCC CCC GGC GCG TGC CTT GGG CTG CGG T
A → G	GGC GGG TCC TGG GTG TCT CCG GGG GGG GGG TGG CTT GGG GTG GGG T
A → T	GGC TGT TCC TGT TTT TCT CCT TTT GGT GTG TGT CTT GGG TTG TGG T
C → A	GGA AGA TAA TGA ATA TAT AAA AAA GGA GAG TGA ATT GGG ATG AGG T
C → G	GGG AGA TGG TGA ATA TGT GGA AAA GGA GAG TGA GTT GGG ATG AGG T
C → T	GGT AGA TTT TGA ATA TTT TTA AAA GGA GAG TGA TTT GGG ATG AGG T
G → A	AAC AAA TCC TAA ATA TCT CCA AAA AAA AAA TAA CTT AAA ATA AAA T
G → C	CCC ACA TCC TCA ATA TCT CCA AAA CCA CAC TCA CTT CCC ATC ACC T
G → T	TTC ATA TCC TTA ATA TCT CCA AAA TTA TAT TTA CTT TTT ATT ATT T
T → A	GGC AGA ACC AGA AAA ACA CCA AAA GGA GAG AGA CAA GGG AAG AGG A
T → C	GGC AGA CCC CGA ACA CCC CCA AAA GGA GAG CGA CCC GGG ACG AGG C
T → G	GC AGA GCC GGA AGA GCG CCA AAA GGA GAG GGA CGG GGG AGG AGG G
GA → GC	GGC AGC TCC TGC ATA TCT CCA AAA GGC GCG TGC CTT GGG CTG CGG T
GG → TG	TGC AGA TCC TGA ATA TCT CCA AAA TGA GAG TGA CTT TTG ATG ATG T
AG → AC	GGC ACA TCC TGA ATA TCT CCA AAA CGA CAC TGA CTT GGG ATG ACG T
TG → TA	GGC AGA TCC TAA ATA TCT CCA AAA GGA GAG TAA CTT AGG ATA AGG T
GG → CG	CGC AGA TCC TGA ATA TCT CCA AAA CGA GAG TGA CTT CCG ATG ACG T
AG → GG	GGC GGA TCC TGA ATA TCT CCG GGG GGG GGG TGA CTT GGG ATG GGG T
CT → AT	GGC AGA TCA TGA ATA TAT CCA AAA GGA GAG TGA ATT GGG ATG AGG T
GG → GT	GTC AGA TCC TGA ATA TCT CCA AAA GTA GAG TGA CTT GTT ATG AGT T
TG → AG	GGC AGA TCC AGA ATA TCT CCA AAA GGA GAG AGA CTA GGG AAG AGG T
TT → TA	GGC AGA TCC TGA ATA TCT CCA AAA GGA GAG TGA CTA GGG ATG AGG T
AT → GT	GGC AGG TCC TGA GTG TCT CCA AAA GGA GAG TGA CTT GGG GTG AGG T
GA → GG	GGC AGG TCC TGG GTA TCT CCA AAA GGG GGG TGG CTT GGG GTG GGG T
Sensor 1 OFF	GGC CGA TCC TGA ATA TCT CCA AAA GGC GCG TGA CTT GGG ATG CGG T
Sensor 2 OFF	CGC AGA TCC TGA ATA TCT CCA AAA CGA GAG TGA CTT CCG ATG ACG T
Sensor 3 OFF	GGC AGA TCC TGA ATA TCT CCA AAA GGA GAG CGA CTC GGG ACG AGG T
Sensor 4 OFF	GGC GGA TCC TGA ATA TCT CCA AAA GGA GAG TGA CTT GGG ATG AGG T
Sensor 5 OFF	GGA AGA AAA AGA ATA TCG CGA AAA GGG GAG TGA CGT GGG ATG GGG G
Sensor 5 ON	GGA AGA AAA AGA ATA TCC CCA AAA GGG GAG TGA CCT GGG ATG GGG G
Sensor 6 OFF	GGC AGA TCC TGA ATA TCG CCA AAA GGA GAG TGA CGT GGG ATG AGG T
Sensor 7 OFF	GGC AGA TCC TGT ATA TCT CCA TCA GAA GAG TAA CTT GGA ATA AGG T
Sensor 7 ON	GGC AGA TCC TGT ATA TCT CCA TCA GGA GAG TGA CTT GGG ATG AGG T
Sensor 8 OFF	GGA AGC AAA AGC ATA TCC CCA AAA GGG GCG TGC CTT GGG CTG GGG G
Sensor 8 ON	GGA AGA AAA AGA ATA TCC CCA AAA GGG GAG TGA CTT GGG ATG GGG G
Sensor 9 OFF	GGA AGA AAA AGA GTG TCC CCA AAA GGG GAA TGA CTT GGG GTG GGG G
Sensor 9 ON	GGA AGA AAA AGA ATA TCC CCA AAA GGG GAA TGA CTT GGG ATG GGG G
Sensor 10 OFF	GGC AGA TCC TGA ATA ATT CCA AAA GGA GTG TGA CTT GGG ATG AGG T
Sensor 10 ON	GGC AGA TCC TGA ATA ACT CCA AAA GGA GTG TGA CCT GGG ATG AGG T
Turn OFF, 4 TC	GGC AGT AAA AGA ATT TCT CTA AAA AGA GAG TGA CTT GGG ATG AGT G
Turn OFF, 4 TdU	GGC AGT AAA AGA ATT T[U]T [U]TA AAA AGA GAG TGA CTT GGG ATG AGT G
Turn OFF, 5 TC	GGC AGA AAA AGA ATA TCT CTA AAA AGA GAG TGA CTT GGG ATG AGT G
Turn OFF, 5 TdU	GGC AGA AAA AGA ATA T[U]T [U]TA AAA AGA GAG TGA CTT GGG ATG AGT G
Turn OFF, 8 TC	GGC AGA AAA AGA ATA TCC CTA AAA AGG GAG TGA CTT GGG ATG GGT G
Turn OFF, 8 TdU	GGC AGA AAA AGA ATA T[U]C CTA AAA AGG GAG TGA CTT GGG ATG GGT G
Turn OFF, 9 TC	GGC AGA AAA AGA ATT TCT CTA AAA AGA GAG TGA CTT GGG ATG AGT G
Turn OFF, 9 TdU	GGC AGA AAA AGA ATT T[U]T [U]TA AAA AGA GAG TGA CTT GGG ATG AGT G
Turn OFF, 11 TC	GGC AGT AAA AGA ATT TCT CCA AAA GGA GAG TGA CTT GGG ATG AGG T
Turn OFF, 11 TdU	GGC AGT AAA AGA ATT T[U]T [U]CA AAA GGA GAG TGA CTT GGG ATG AGG T
Turn OFF, 16 TC	GGC AGA AAA AGA ATA TCT TCA AAA GGA GAG TGA CTT GGG ATG AGG G

Turn OFF, 16 TdU	GGC AGA AAA AGA ATA T[U]T T[U]A AAA GGA GAG TGA CTT GGG ATG AGG G
Turn OFF, 23 TC	GGC AGA AAA AGA ATT TCT CCA AAA GGA GAG TGA CTT GGG ATG AGG T
Turn OFF, 23 TdU	GGC AGA AAA AGA ATT T[U]T [U]CA AAA GGA GAG TGA CTT GGG ATG AGG T
Turn OFF, 26 TC	GGC AGA AAA ATG AAT TTC CCA AAA GGG GAG TGA CTT GGG ATG GGG T
Turn OFF, 26 TdU	GGC AGA AAA ATG AAT TT[U] CCA AAA GGG GAG TGA CTT GGG ATG GGG T
Turn OFF, 30 TC	GGC AGT AAA AGA ATT TCT CCA AAA GGA GAG TGA CTT GGG ATG AGG G
Turn OFF, 30 TdU	GGC AGT AAA AGA ATT T[U]T [U]CA AAA GGA GAG TGA CTT GGG ATG AGG G
Turn OFF, 40 TC	GGC AGA AAA AGA ATA TCT CCA AAA GGA GAG TGA CTT GGG ATG AGG G
Turn OFF, 40 TdU	GGC AGA AAA AGA ATA T[U]T [U]CA AAA GGA GAG TGA CTT GGG ATG AGG G
Turn OFF, 52 TC	GGC AGT AAA AGA ATA TCT CTA AAA AGA GAG TGA CTT GGG ATG AGT C
Turn OFF, 52 TdU	GGC AGT AAA AGA ATA T[U]T [U]TA AAA AGA GAG TGA CTT GGG ATG AGT C
Turn OFF, 53 TC	GGC AGA AAA AGA ATA TCT CTA AAA AGA GAG TGA CTT GGG ATG AGA T
Turn OFF, 53 TdU	GGC AGA AAA AGA ATA T[U]T [U]TA AAA AGA GAG TGA CTT GGG ATG AGA T
Turn OFF, 60 TC	GGC AGA AAA AGA ATA TCT CTA AAA AGA GAG TGA CTT GGG ATG AGT C
Turn OFF, 4 60dU	GGC AGA AAA AGA ATA T[U]T [U]TA AAA AGA GAG TGA CTT GGG ATG AGT C
Turn OFF, 196 TC	GGC AGT AAA AGA ATT TCT CTA AAA AGA GAG TGA CTT GGG ATG AGT C
Turn OFF, 196 TdU	GGC AGT AAA AGA ATT T[U]T [U]TA AAA AGA GAG TGA CTT GGG ATG AGT C
Turn ON, 7 TC	GGC AGA TCC TCA ATA TCC CCA AAA GGG GAG TGA CTT GGG ATG GGG T
Turn ON, 7 TdU	GGC AGA TCC T[U]AA TAT CCC CAA AAG GGG AGT GAC TTG GGA TGG GGT
Turn ON, 11 TC	GGC AGA TCC TGA ATC TCC CCA AAA GGG GAG TGA CTT GGG ATG GGG T
Turn ON, 11 TdU	GGC AGA TCC TGA AT[U] TCC CCA AAA GGG GAG TGA CTT GGG ATG GGG T
Turn ON, 27 TC	GGC AGA TCC TGA ATA TCC CCA AAA GGG GAG TGA CCT GGG ATG GGG T
Turn ON, 27 TdU	GGC AGA TCC TGA ATA TCC CCA AAA GGG GAG TGA C[U]T GGG ATG GGG T
Turn ON, 28 TC	GGC AGA TCC TGA ATA TCC CCA AAA GGG GAG TGA CTC GGG ATG GGG T
Turn ON, 28 TdU	GGC AGA TCC TGA ATA TCC CCA AAA GGG GAG TGA CT[U] GGG ATG GGG T
Turn ON, 11/28 TC	GGC AGA TCC TGA ATC TCC CCA AAA GGG GAG TGA CTC GGG ATG GGG T
Turn ON, 11/28 TdU	GGC AGA TCC TGA AT[U] TCC CCA AAA GGG GAG TGA CT[U] GGG ATG GGG T
Turn ON mut 1, CGT	GGC AGA TCC TGA AT[U] TCC CCA AAA GGG GAG TGA CT[U] GGG ATG TGG T
Turn ON mut 2, CAG	GGC AGA TCC TGA AT[U] TCC CCA AAA GGA GAG TGA CT[U] GGG ATG GGG T
Turn ON mut 3, TGG	GGC AGA TCC TGA AT[U] TCT CCA AAA GGG GAG TGA CT[U] GGG ATG GGG T
Turn ON mut 4, TAG	GGC AGA TCC TGA AT[U] TCT CCA AAA GGA GAG TGA CT[U] GGG ATG GGG T
Turn ON mut 5, TGT	GGC AGA TCC TGA AT[U] TCT CCA AAA GGG GAG TGA CT[U] GGG ATG TGG T
Turn ON mut 6, CAT	GGC AGA TCC TGA AT[U] TCC CCA AAA GGA GAG TGA CT[U] GGG ATG TGG T
Turn ON restored	GGC AGA TCC TGA AT[U] TCT CCA AAA GGA GAG TGA CT[U] GGG ATG TGG T
Turn OFF mut 1, TAG	GGC AGT AAA AGA ATT TCT CTA AAA AGA GAG TGA CTT GGG ATG GGT T
Turn OFF mut 2, TGT	GGC AGT AAA AGA ATT TCT CTA AAA AGG GAG TGA CTT GGG ATG TGT T
Turn OFF mut 3, CAT	GGC AGT AAA AGA ATT TCC CTA AAA AGA GAG TGA CTT GGG ATG TGT T
Turn OFF mut 4, CGT	GGC AGT AAA AGA ATT TCC CTA AAA AGG GAG TGA CTT GGG ATG TGT T
Turn OFF mut 5, CAG	GGC AGT AAA AGA ATT TCC CTA AAA AGA GAG TGA CTT GGG ATG GGT T
Turn OFF mut 6, TGG	GGC AGT AAA AGA ATT TCT CTA AAA AGG GAG TGA CTT GGG ATG GGT T
Turn OFF restored	GGC AGT AAA AGA ATT TCC CTA AAA AGG GAG TGA CTT GGG ATG GGT T
Turn ON, 11/28, best	GGC AGA TCC TGA ATC CCC CCA AAA GGG GGG TGA CTC GGG ATG GGG T

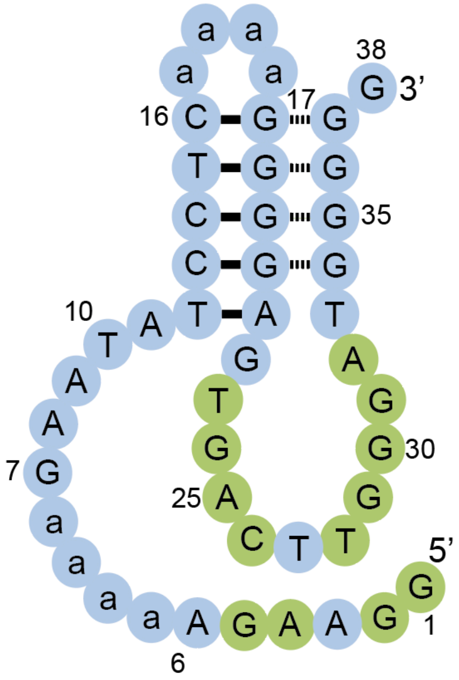

Figure S1. Conserved positions in Supernova.
Secondary structure model of the parental sequence of Supernova as described in our previous work [13]. The most highly conserved positions are highlighted in green. Positions with bases shown in lowercase font correspond to those in AAAA spacers that were introduced in minimization experiments, and are not numbered.

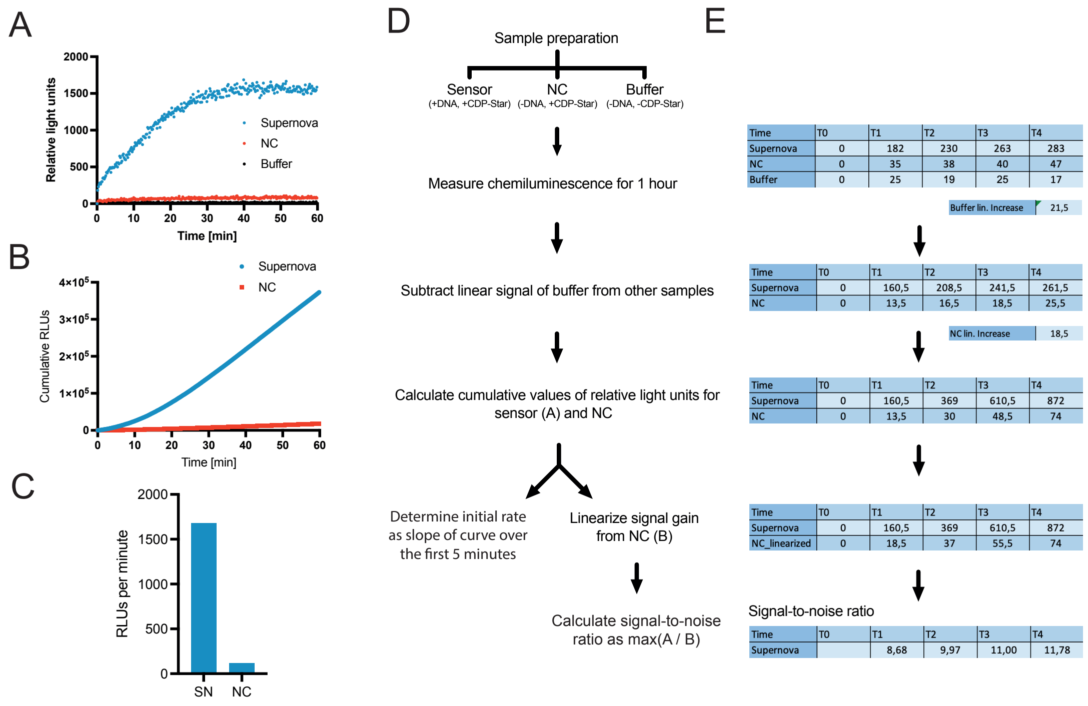

Figure S2. Analysis of light-producing reactions.
A) Representative time course showing light production as a function of time. B) Representative time course showing total light production as a function of time. C) Representative calculation of initial rates of light production for catalyzed and non-catalyzed reactions for data shown in panel B. D) Workflow of analysis. E) Example of data analysis using the workflow shown in panel D. This type of analysis was used to calculate total light production (as shown in Figures 6 and 7), RLUs per minute (as shown in Figures 2, 3, 6, and 7) and signal to noise ratios for sensors characterized in this study.

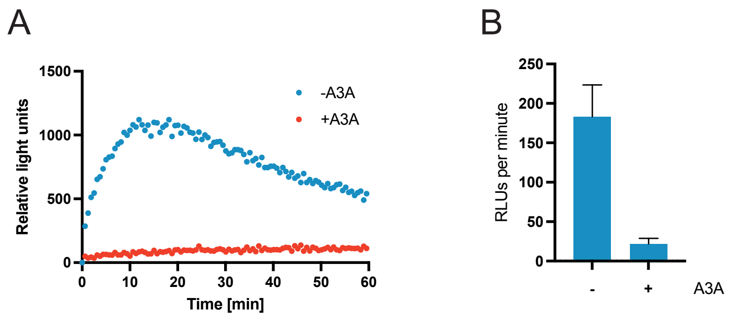

Figure S3. Light production of a turn-off sensor. A) Time course of light production of a turn-off sensor in the absence (blue dots) or presence (red dots) of APOBEC3A. Reactions containing 3.3 µM deoxyribozyme and 50 nM APOBEC3A were incubated in a buffer containing 20 mM NaCl, 0.5% Triton, and 7.5 mM Tris-HCl pH 7.4 for 20 minutes. After adding CDP-Star and Supernova buffer (final concentrations = 1 µM deoxyribozyme, 15 nM APOBEC3A, 62.5 µM CDP-Star, 650 µM $ZnCl_2$, 20 mM KCl, and 20 mM Tris-HCl pH 7.4, as well as diluted components from the previous buffer), reactions were put into a plate reader, and cumulative light production was measured for one hour. B) Initial rates of light production of a turn-off sensor preincubated in the absence or presence of APOBEC3A.

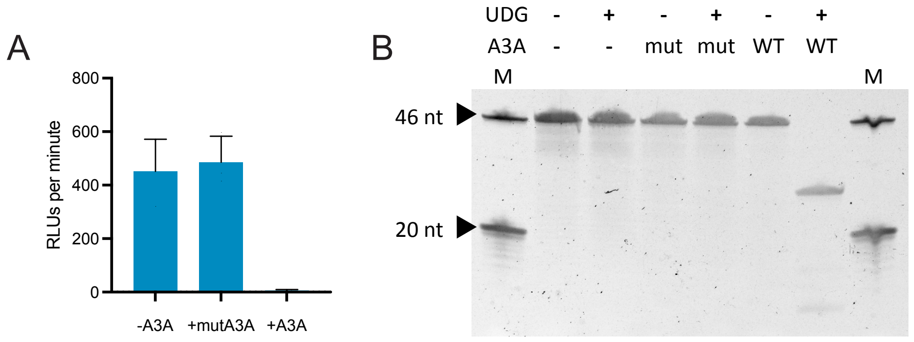

Figure S4. Inactivation of the turn-off sensor requires catalytically active APOBEC3A, and induces C to U changes in the sequence of the sensor. A) Initial rate of light production of the turn-off sensor preincubated in the absence of APOBEC3A, in the presence of catalytically inactive APOBEC3A, or in the presence of catalytically active APOBEC3A. Reactions containing 3.3 µM deoxyribozyme and 0 or 50 nM of either mutated APOBEC3A (E72A), or wild-type APOBEC3A were incubated in a buffer containing 20 mM NaCl, 0.5% Triton, and 7.5 mM Tris-HCl pH 7.4 for 20 minutes. After adding CDP-Star and Supernova buffer (final concentrations = 1 µM deoxyribozyme, 15 nM APOBEC3A, 62.5 µM CDP-Star, 650 µM $ZnCl_2$, 20 mM KCl, and 20 mM Tris-HCl pH 7.4, as well as diluted components from the previous buffer), reactions were put into a plate reader. Cumulative light production was measured for one hour, and the initial rate of light production was determined from time points in the first 5 minutes of each reaction. B) Analysis of C to U changes in the sensor induced by APOBEC3A using the UDG assay. Reactions containing 3.3 µM deoxyribozyme and 0 or 50 nM of either mutated APOBEC3A (E72A), or wild-type APOBEC3A were incubated in a buffer containing 20 mM NaCl, 0.5% Triton, and 7.5 mM Tris-HCl pH 7.4 for 20 minutes. The reaction was stopped by adding 1 mM EDTA and 1 µl of 1M Tris pH 8.5. UDG was added at a concentration of 1 U/reaction and samples were incubated at 37 °C for 30 minutes. Debased sites were cleaved under basic conditions after adding 100 mM NaOH and an incubation at 80 °C for 20 minutes. Samples were then separated by PAGE on a 15% denaturing gel.

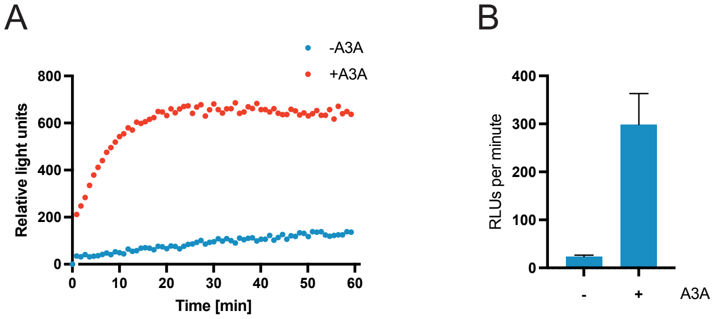

Figure S5. Light production of a turn-on sensor. A) Time course of light production of a turn-on sensor in the absence (blue dots) or presence (red dots) of APOBEC3A. Reactions containing 3.3 µM deoxyribozyme and 50 nM APOBEC3A were incubated in a buffer containing 20 mM NaCl, 0.5% Triton, and 7.5 mM Tris-HCl pH 7.4 for 20 minutes. After adding CDP-Star and Supernova buffer (final concentrations = 1 µM deoxyribozyme, 15 nM APOBEC3A, 62.5 µM CDP-Star, 650 µM $ZnCl_2$, 20 mM KCl, and 20 mM Tris-HCl pH 7.4, as well as diluted components from the previous buffer), reactions were put into a plate reader, and cumulative light production was measured for one hour. B) Initial rates of light production of a turn-on sensor preincubated in the absence or presence of APOBEC3A.

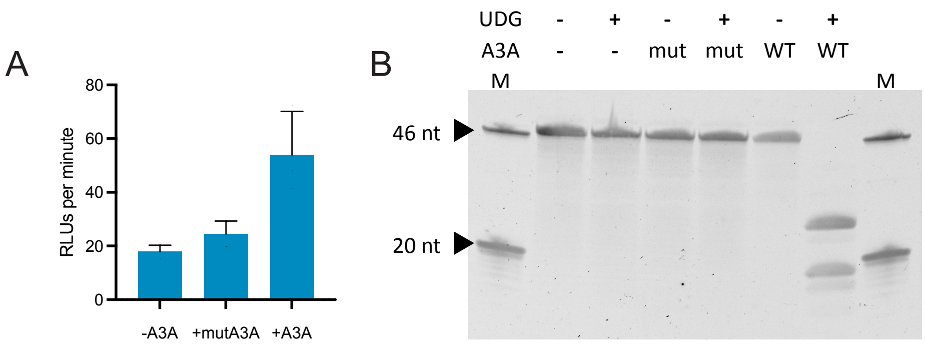

Figure S6. Activation of the turn-on sensor requires catalytically active APOBEC3A, and induces C to U changes in the sequence of the sensor. A) Initial rate of light production of the turn-on sensor preincubated in the absence of APOBEC3A, in the presence of catalytically inactive APOBEC3A, or in the presence of §catalytically active APOBECA. Reactions containing 3.3 µM deoxyribozyme and 0 or 50 nM of either mutated APOBEC3A (E72A), or wild-type APOBEC3A were incubated in a buffer containing 20 mM NaCl, 0.5% Triton, and 7.5 mM Tris-HCl pH 7.4 for 20 minutes. After adding CDP-Star and Supernova buffer (final concentrations = 1 µM deoxyribozyme, 15 nM APOBEC3A, 62.5 µM CDP-Star, 650 µM $ZnCl_2$, 20 mM KCl, and 20 mM Tris-HCl pH 7.4, as well as diluted components from the previous buffer), reactions were put into a plate reader. Cumulative light production was measured for one hour, and the initial rate of light production was determined from time points in the first 5 minutes of each reaction. B) Analysis of C to U changes in the sensor induced by APOBEC3A using the UDG assay. Reactions containing 3.3 µM deoxyribozyme and 0 or 50 nM of either mutated APOBEC3A (E72A), or wild-type APOBEC3A were incubated in a buffer containing 20 mM NaCl, 0.5% Triton, and 7.5 mM Tris-HCl pH 7.4 for 20 minutes. The reaction was stopped by adding 1 mM EDTA and 1 µl of 1M Tris pH 8.5. UDG was added at a concentration of 1 U/reaction and samples were incubated at 37 °C for 30 minutes. Debased sites were cleaved under basic conditions after adding 100 mM NaOH and an incubation of 80 °C for 20 minutes. Samples were then separated by PAGE on a 15% denaturing gel.

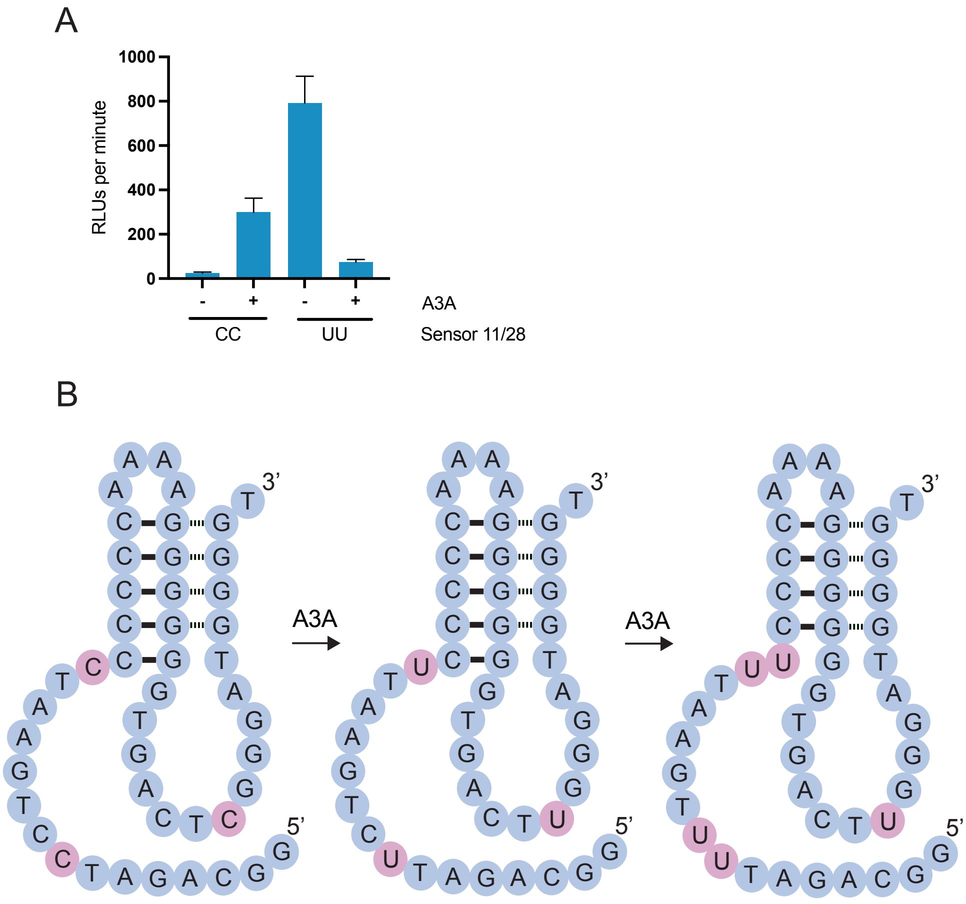

Figure S7. Inhibitory chain reaction of APOBEC3A. A) Light production of a turn-on sensor containing TC motifs and its counterpart in which these have been replaced by TU motifs (corresponding to the expected product of the APOBEC3A editing reaction). While the TC version of this sensor is activated by APOBEC3A, the TU version is inhibited by APOBEC3A. B) Possible model of inhibition. A deaminated cytosine is read by APOBEC3A as thymine. If this is followed by a second cytosine, it creates a new editing site and can result in a second editing reaction. Reactions containing 3.3 µM deoxyribozyme and 50 nM APOBEC3A were incubated in a buffer containing 20 mM NaCl, 0.5% Triton, and 7.5 mM Tris-HCl pH 7.4 for 20 minutes. After adding CDP-Star and Supernova buffer (final concentrations = 1 µM deoxyribozyme, 15 nM APOBEC3A, 62.5 µM CDP-Star, 650 µM $ZnCl_2$, 20 mM KCl, and 20 mM Tris-HCl pH 7.4, as well as diluted components from the previous buffer), reactions were put into a plate reader. Cumulative light production was measured for one hour, and the initial rate of light production was determined from time points in the first 5 minutes of each reaction.

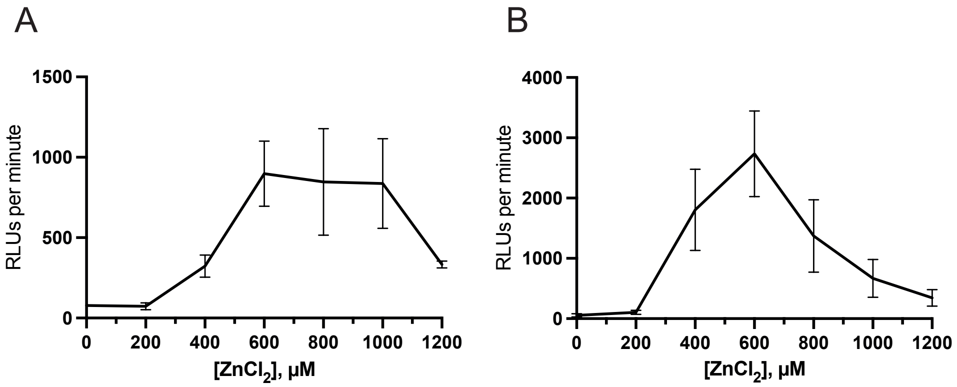

Figure S8. Zinc dependence of APOBEC3A sensors. A) Turn-off sensor. B) Turn-on sensor. Reactions contained 1 µM deoxyribozyme and 62.5 µM CDP-Star in a buffer containing varying concentrations of $ZnCl_2$, 20 mM KCl, and 20 mM Tris-HCl pH 7.4. Cumulative light production was measured for one hour, and the initial rate of light production was determined from time points in the first 5 minutes of each reaction.

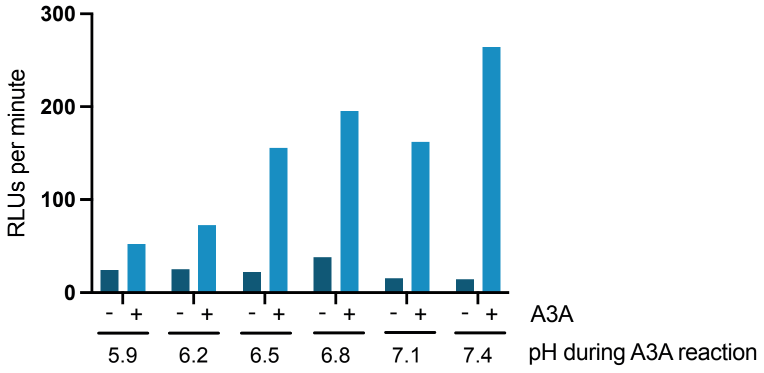

Figure S9. pH dependence of an APOBEC3A turn-on sensor. Reactions containing 3.3 µM deoxyribozyme and 50 nM APOBEC3A were incubated in a buffer containing 20 mM NaCl, 0.5% Triton, and 7.5 mM MES or Tris-HCl, pH ranging from 5.9 to 7.4 for 20 minutes. After adding CDP-Star and Supernova buffer (final concentrations = 1 µM deoxyribozyme, 15 nM APOBEC3A, 62.5 µM CDP-Star, 650 µM $ZnCl_2$, 20 mM KCl, and 20 mM Tris-HCl pH 7.4, as well as diluted components from the previous buffer), reactions were put into a plate reader. Cumulative light production was measured for one hour, and the initial rate of light production was determined from time points in the first 5 minutes of each reaction.

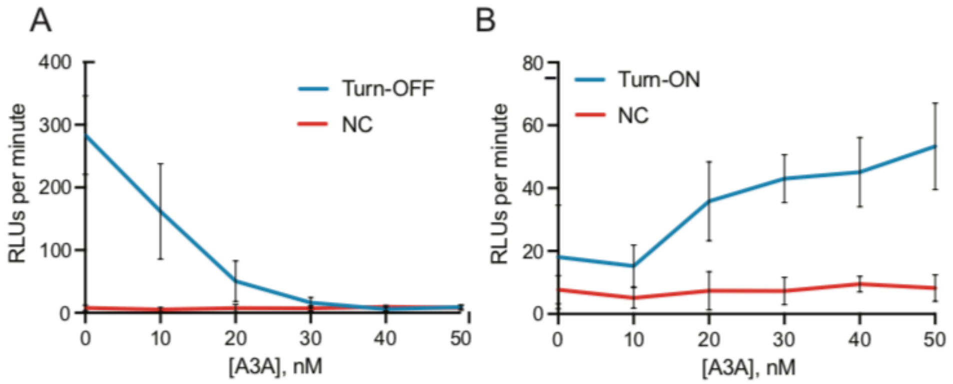

Figure S10. APOBEC3A concentration dependence. A) Turn-off sensor. B) Turn-on sensor. Reactions containing 3.3 μM deoxyribozyme and the indicated concentration of APOBEC3A were incubated in a buffer containing 20 mM NaCl, 0.5% Triton, and 7.5 mM Tris-HCl pH 7.4 for 20 minutes. After adding CDP-Star and Supernova buffer (final concentrations = 1 μM deoxyribozyme, varying concentrations of APOBEC3A, 62.5 μM CDP-Star, 650 μM $ZnCl_2$, 20 mM KCl, and 20 mM Tris-HCl pH 7.4, as well as diluted components from the previous buffer), reactions were put into a plate reader. Cumulative light production was measured for one hour, and the initial rate of light production was determined from time points in the first 5 minutes of each reaction.

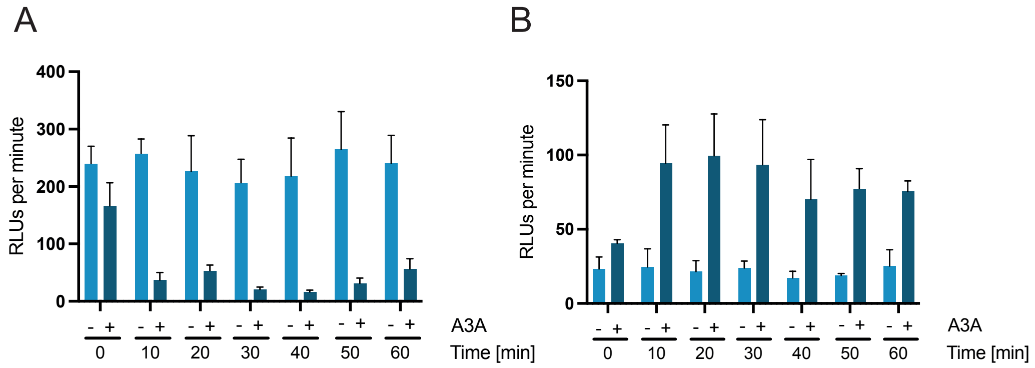

Figure S11. APOBEC3A incubation time dependence. A) Turn-off sensor. B) Turn-on sensor. Reactions containing 3.3 μM deoxyribozyme and 50 nM APOBEC3A were incubated in a buffer containing 20 mM NaCl, 0.5% Triton, and 7.5 mM Tris-HCl pH 7.4 for the indicated time. After adding CDP-Star and Supernova buffer (final concentrations = 1 μM deoxyribozyme, 15 nM APOBEC3A, 62.5 μM CDP-Star, 650 μM $ZnCl_2$, 20 mM KCl, and 20 mM Tris-HCl pH 7.4, as well as diluted components from the previous buffer), reactions were put into a plate reader. Cumulative light production was measured for one hour, and the initial rate of light production was determined from time points in the first 5 minutes of each reaction.

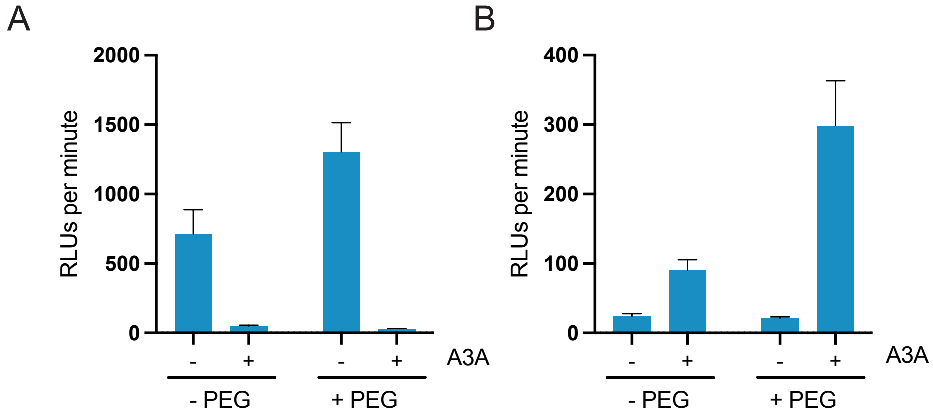

Figure S12. Effect of PEG on APOBEC3A sensors. A) Turn-off sensor. B) Turn-on sensor. Reactions containing 3.3 µM deoxyribozyme and 50 nM APOBEC3A were incubated in a buffer containing 20 mM NaCl, 0.5% Triton, and 7.5 mM Tris-HCl pH 7.4 for 20 minutes. After adding CDP-Star and Supernova buffer (final concentrations = 1 µM deoxyribozyme, 15 nM APOBEC3A, 62.5 µM CDP-Star, 650 µM $ZnCl_2$, 20 mM KCl, 20 mM Tris-HCl pH 7.4, and either 0% or 20% V/V PEG 200, as well as diluted components from the previous buffer), reactions were put into a plate reader. Cumulative light production was measured for one hour, and the initial rate of light production was determined from time points in the first 5 minutes of each reaction.

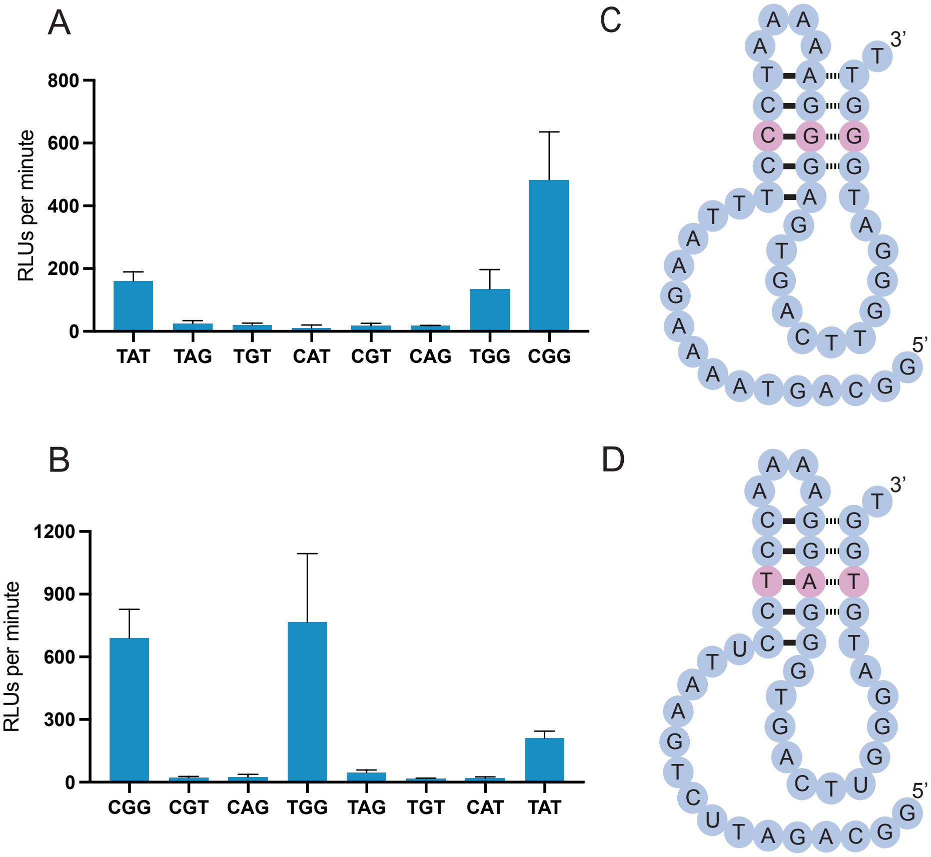

Figure S13. Evidence for a triple helix in turn-off and turn-on sensors. A) Disruption and mutational rescue of a proposed base triple in a turn-off sensor. B) Same, but for a turn-on sensor. C) Secondary structure model of the most active version of the turn-off sensor shown in panel A, with the mutated base triple shown in pink. D) Same, but for the turn-on sensor shown in panel B. Reactions containing 1 µM deoxyribozyme, 62.5 µM CDP-Star, 650 µM $ZnCl_2$, 20 mM KCl, and 20 mM Tris-HCl pH 7.4 were put into a plate reader. Cumulative light production was measured for one hour, and the initial rate of light production was determined from time points in the first 5 minutes of each reaction.
